# Artificial Intelligence Electrocardiogram-Derived Heart Age Predicts Long-Term Mortality After Transcatheter Aortic Valve Replacement

**DOI:** 10.1016/j.jacadv.2024.101171

**Published:** 2024-08-21

**Authors:** Ghasaq Saleh, Agata Sularz, Chia-Hao Liu, Gerardo V. Lo Russo, Mahmoud Zhour Adi, Zachi Attia, Paul Friedman, Rajiv Gulati, Mohamad Alkhouli

**Affiliations:** Department of Cardiovascular Medicine, Mayo Clinic, Rochester, Minnesota, USA

**Figure:**
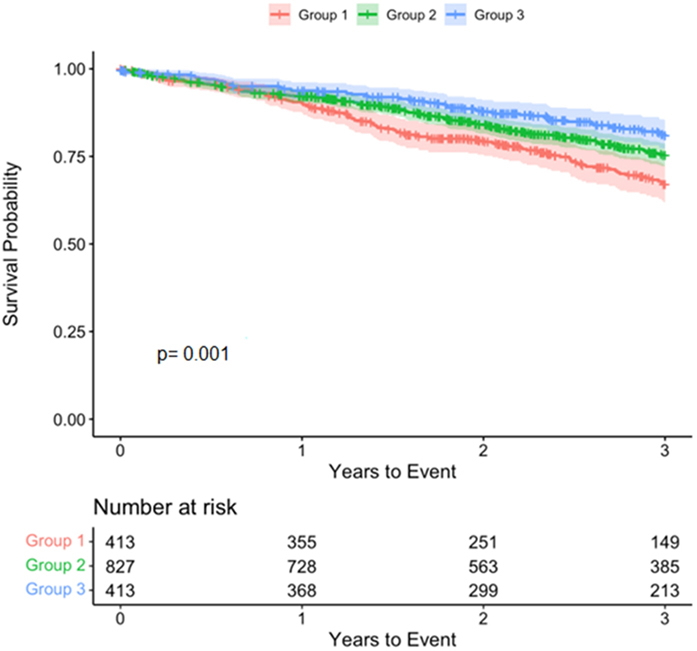

Age significantly influences the risk of cardiovascular (CV) disease. However, chronological age may not always align with CV age, a disparity referred to as the “age gap.” Various methods, including genetic, cellular, and biochemical techniques, have been utilized to estimate CV age. Recent advancements in artificial intelligence (AI) algorithms enable the estimation of CV age through deep analysis of 12-lead electrocardiogram (ECG).^1^^,^^2^ The AI-ECG-derived age gap was found to be predictive of clinical outcomes in heart and renal transplant patients.^3^^,^^4^ In this focused analysis, we sought to explore whether the AI-ECG-derived age gap is associated with long-term mortality after transcatheter aortic valve replacement (TAVR).

We retrospectively studied patients who underwent TAVR at Mayo Clinic, Rochester, MN, between September 2011 and November 2023. AI-ECG age was estimated using Mayo Clinic’s AI ECG-heart age algorithm described and validated elsewhere.^2^ Patients with existing pacemakers and those with missing preprocedural ECGs were excluded. Also, patients >90 years of age were excluded, given that the original training dataset did not include nonagenarians.^2^ The age gap was calculated as the *difference between chronological age at the time of the most immediate preprocedural ECG and AI-ECG heart age*. The primary outcome was all-cause mortality. The study was approved by the institutional review board.

Patients were stratified into 3 groups according to the age gap (group 1: <25th percentile; group 2: 25–75th percentile; group 3: >75th percentile); group 1 (age gap <25th percentile) included patients who had an AI-ECG age that was greater than their chronological age or, their chronological age was greater than AI ECG age by no more than 1.78 years. Group 3 (age gap >75th percentile) had an AI-ECG age that was always less than their chronological age (the difference of at least 11.3 years between the two).

Survival analysis was performed using a log-rank test with Kaplan-Meier curves to determine the difference in primary outcome between groups. Baseline characteristics were compared between the groups using Student’s t-test or the Mann-Whitney U test for continuous variables and Pearson’s chi-square for categorical variables. Adjusted HR were calculated using a Cox-proportional hazards regression model and were depicted with their 95% CI. The overall model adjusted for potential confounders and group differences in baseline characteristics with a *P* value of <0.05.

**Figure 1:**
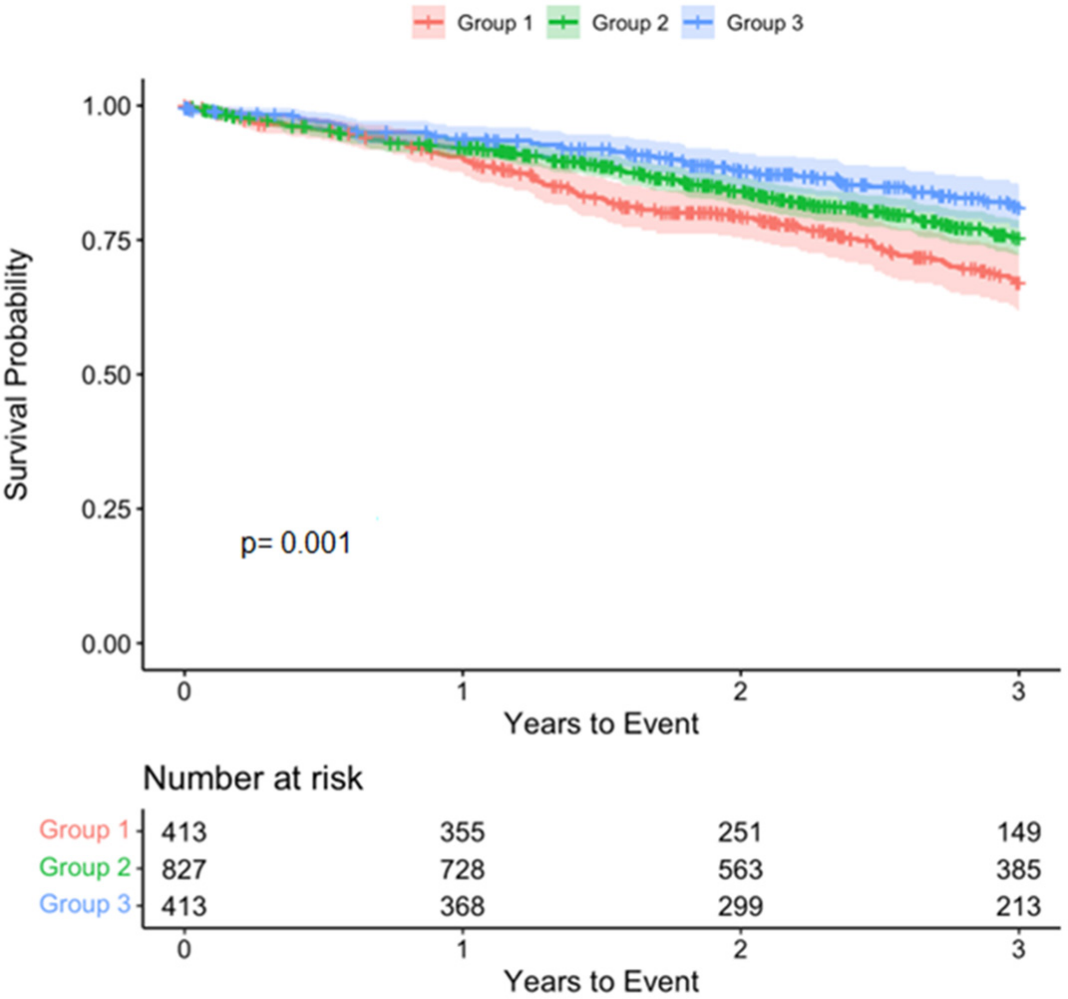
Survival Analysis Illustration of the Association Between Age Gap (Difference Between Chronological Age and AI-ECG Heart Age) and All-Cause Mortality After Transcatheter Aortic Valve Replacement Patients were stratified according to their age gap: group 1: <25th percentile, group 2: 25-75th percentile, group 3: >75th percentile. AI = artificial intelligence; ECG = electrocardiogram

A total of 1,653 patients were included (58.9% men). The mean chronological and AI-ECG ages were 78.3 ± 7.7 years and 72.3 ± 7.1 years, respectively. The mean age gap was 6.0 ± 7.4 years. In-hospital mortality was 0.3%. The median follow-up duration was 2.8 years. The 25th and 75th percentile values for the age gap were 1.78 and 11.3 years, respectively. The cumulative 3-year survival was 62% in group 1, 77% in group 2, and 81% in group 3 (*P* < 0.001) (Figure 1). In the Cox proportional hazards models, the patients in group 1 were at a higher risk of all-cause mortality (HR: 1.64; 95% CI: 1.12-2.40; *P* = 0.011) compared to patients in groups 2 and 3. Age gap as a continuous variable was also significantly associated with all-cause mortality in both the univariable (HR: 0.97; 95% CI: 0.95-0.98; *P* < 0.001) and multivariable models (HR: 0.97; 95% CI: 0.96-0.99; *P* = 0.003). Other variables that were included in the multivariable Cox model and were associated with increased risk of all-cause mortality included atrial fibrillation, chronic lung disease, porcelain aorta, dialysis status, weight, body mass index, and albumin.

To our knowledge, this is the first study that assessed the role of AI-ECG-derived heart age in forecasting outcomes of transcatheter interventions. Our study shows that in patients undergoing TAVR, patients who are “older” by ECG than their stated chronological age experienced a higher all-cause mortality at 3 years after the procedure. The association of the “age gap” with mortality aligns with previous findings in other cohorts. In an undifferentiated cohort of 25,000 healthy participants, the age gap had a significant association with increased risk of mortality.^1^ Similarly, in patients waitlisted for renal transplant, a 10-year increase in age gap was associated with an increased risk of mortality (HR: 3.59; 95% CI: 2.06-5.72; *P* < 0.001).^4^ In patients undergoing heart transplant, an increase in AI-ECG heart age post-transplant was associated with higher rates of major adverse events (HR: 1.58; 95% CI: 1.19-2.10; *P* = 0.002).^3^

Our study has some limitations. The AI-ECG heart age model was trained using ECG from a younger population (age 58.6 ± 16.2 years) and not specifically from TAVR patients. However, the sample size of TAVR patients precludes training a dedicated algorithm for physiological age estimation in this specific population. The strong association between AI ECG-derived heart age and post-TAVR mortality documented in this study serves as hypothesis-generating. It is likely that AI-ECG age captures key aging and frailty indicators not evident in traditional risk models, which contributed to the positive findings. However, future studies are needed to validate these findings and assess the value of AI-ECG age in the context of other valve diseases.
